# A novel technique using ultrasonography in upper airway management after anterior cervical decompression and fusion

**DOI:** 10.1186/s12880-022-00792-8

**Published:** 2022-04-12

**Authors:** Shizumasa Murata, Hiroshi Iwasaki, Hiroyuki Oka, Hiroshi Hashizume, Yasutsugu Yukawa, Akihito Minamide, Shunji Tsutsui, Masanari Takami, Keiji Nagata, Ryo Taiji, Takuhei Kozaki, Hiroshi Yamada

**Affiliations:** 1 Department of Orthopedic Surgery, Wakayama Medical University, 811-1 Kimiidera, Wakayama City, Wakayama 641-8510 Japan; 2 Department of Medical Research and Management for Musculoskeletal Pain, 22nd Century Medical & Research Center, The University of Tokyo Hospital, Hongo 7-3-1, Bunkyo-ku, Tokyo 113-8655 Japan; 3 Spine Center, Dokkyo Medical University Nikko Medical Center, 632 Takatoku, Nikko City, Tochigi 321-2593 Japan

**Keywords:** Anterior cervical decompression and fusion, Ultrasonography, Prevertebral soft tissue evaluation, Cervical spine, Spine surgery, Airway complication

## Abstract

**Background:**

Airway complications are the most serious complications after anterior cervical decompression and fusion (ACDF) and can have devastating consequences if their detection and intervention are delayed. Plain radiography is useful for predicting the risk of dyspnea by permitting the comparison of the prevertebral soft tissue (PST) thickness before and after surgery. However, it entails frequent radiation exposure and is inconvenient. Therefore, we aimed to overcome these problems by using ultrasonography to evaluate the PST and upper airway after ACDF and investigate the compatibility between X-ray and ultrasonography for PST evaluation.

**Methods:**

We included 11 radiculopathy/myelopathy patients who underwent ACDF involving C5/6, C6/7, or both segments. The condition of the PST and upper airway was evaluated over 14 days. The Bland–Altman method was used to evaluate the degree of agreement between the PST values obtained using radiography versus ultrasonography. The Pearson correlation coefficient was used to determine the relationship between the PST measurement methods. Single-level and double-level ACDF were performed in 8 and 3 cases, respectively.

**Results:**

PST and upper airway thickness peaked on postoperative day 3, with no airway complications. The Bland–Altman bias was within the prespecified clinically nonsignificant range: 0.13 ± 0.36 mm (95% confidence interval 0.04–0.22 mm). Ultrasonography effectively captured post-ACDF changes in the PST and upper airway thickness and detected airway edema.

**Conclusions:**

Ultrasonography can help in the continuous assessment of the PST and the upper airway as it is simple and has no risk of radiation exposure risk. Therefore, ultrasonography is more clinically useful to evaluate the PST than radiography from the viewpoint of invasiveness and convenience.

## Background

Airway complications due to the formation of retropharyngeal hematomas or prevertebral soft tissue swelling (PSTS) are the most serious complications following anterior cervical decompression and fusion (ACDF). Airway complications can lead to respiratory distress and airway obstruction, requiring emergency reintubation or tracheostomy [1, 2]. They occur in 2% of ACDF cases and most frequently between 24 and 48 h after surgery [2–4].

Risk factors for postoperative airway complications include the involvement of > 3 operated segments, intraoperative blood loss > 300 mL, and operation time > 90 min [5, 6]. When risk factors are present, surgeons traditionally monitor the condition of the prevertebral soft tissue (PST) by examining cervical spine lateral radiographs taken from postoperative day 1 to day 10 [7, 8]. However, radiographic evaluation is inconvenient and unsafe as it requires transporting the patient or the use of a portable radiography device and exposes the patient to radiation [9].

These problems could be circumvented by using an ultrasound device to evaluate the PST and upper airway after ACDF. Ultrasound can be quickly performed at the bedside, and patients are not exposed to radiation. It allows physicians to safely and accurately carry out various medical procedures [10, 11] and rapidly assess the target anatomy in operating theaters, intensive care units, emergency departments, and even in remote environments.

The use of ultrasound in musculoskeletal medicine has expanded rapidly over the last 2 decades. In the field of soft tissue pathology, diagnostic ultrasound has been widely adopted to monitor synovitis in patients with inflammatory joint disorders, tendinopathy, or traumatic tendon ruptures and to detect intra-articular effusions [12, 13]. Despite its widespread use and its potential in aiding the early recognition and management of airway complications, ultrasound is not routinely used to examine the PST and upper airway after ACDF.

This report describes an ultrasound procedure of evaluating the PST and upper airway. It also describes the compatibility between radiography and ultrasonography for PST evaluation. A representative case and a pilot study are presented.

## Methods

### Patient population of the pilot study

The study protocol was approved by the Research Ethics Committee of Wakayama Medical University (approval number: 3244). All patient-related procedures performed in this study were in accordance with the ethical standards of the Research Ethics Committee of Wakayama Medical University and the 1964 Declaration of Helsinki and its later amendments. Informed consent was obtained from all participants. Eleven patients who underwent ACDF involving 1 or 2 segments (C5/6, C6/7, or both) for the treatment of radiculopathy or myelopathy were prospectively enrolled in this study between January 1, and June 30, 2020. We used a zero-profile cage for all cases. The exclusion criteria were as follows: fusions involving > 3 segments; previous revision surgery or corpectomy; surgically treated trauma, infections, or tumors; and presence of general metabolic diseases, such as rheumatoid arthritis and diabetes, and chronic heart and renal diseases.

### Ultrasound evaluation procedure

**Fig. 1:**
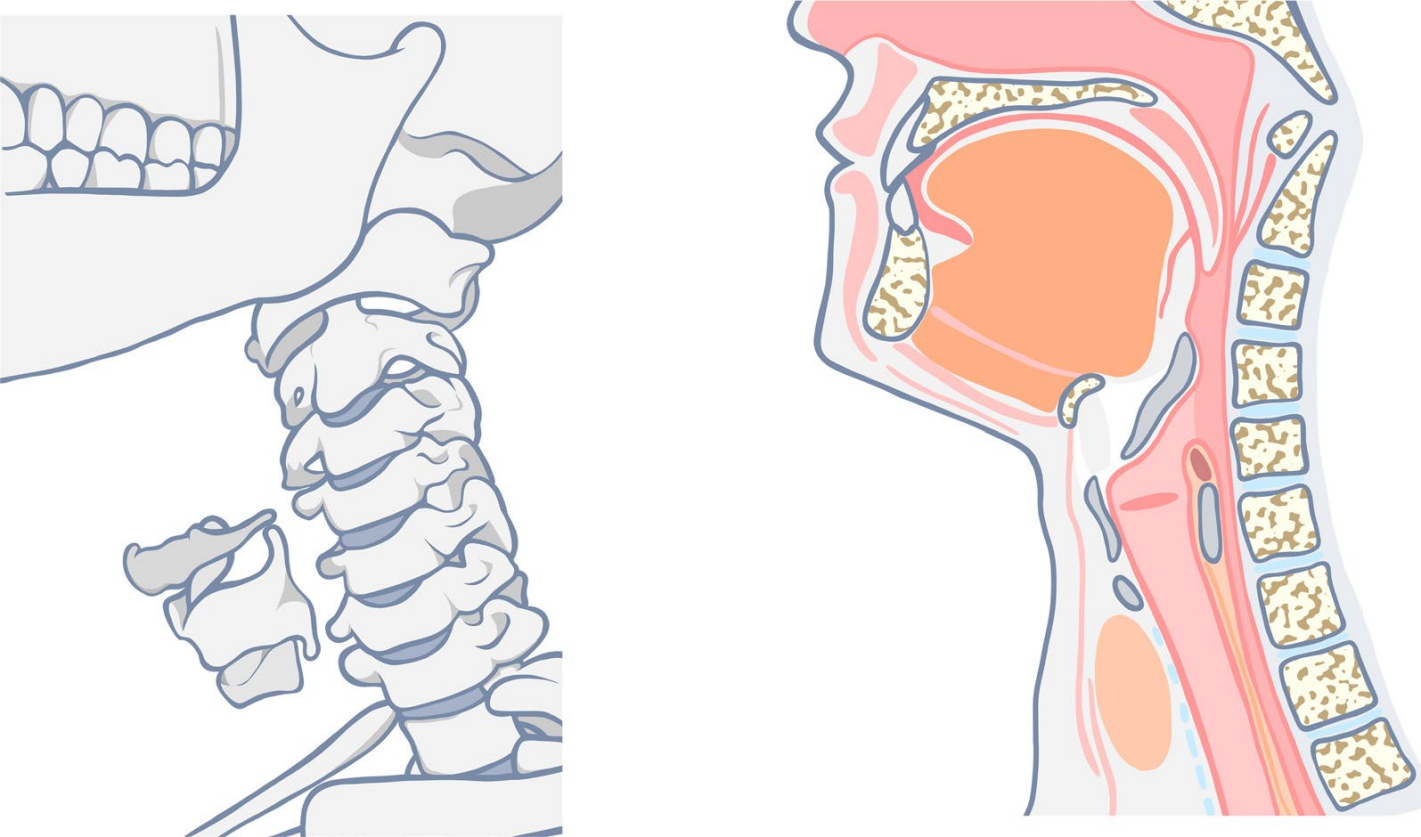
Illustration of the anatomical features around the cervical spine Left (**A**): bones around cervical spine at C5/6. Right (**B**): soft tissues. a: hyoid bone, b: thyroid cartilage, c: cricoid cartilage

Upper airway ultrasound was performed with the patients in the supine position. No special device was required; a standard ultrasound device (SNiBLE; Konica Minolta, Tokyo, Japan) and high-frequency linear probe (L11-3; Konica Minolta) provided sufficient visualization. We identified the desired cervical spine level and tissue by examining the palpable laryngeal prominence of the thyroid cartilage in the cervical region and the palpable hard and smooth cricoid cartilage on the caudal side, which indicate the height of the 5th and 6th cervical vertebral body, respectively (Fig. 1).

### Thyroid cartilage and cricoid cartilage in the transverse plane

**Fig. 2:**
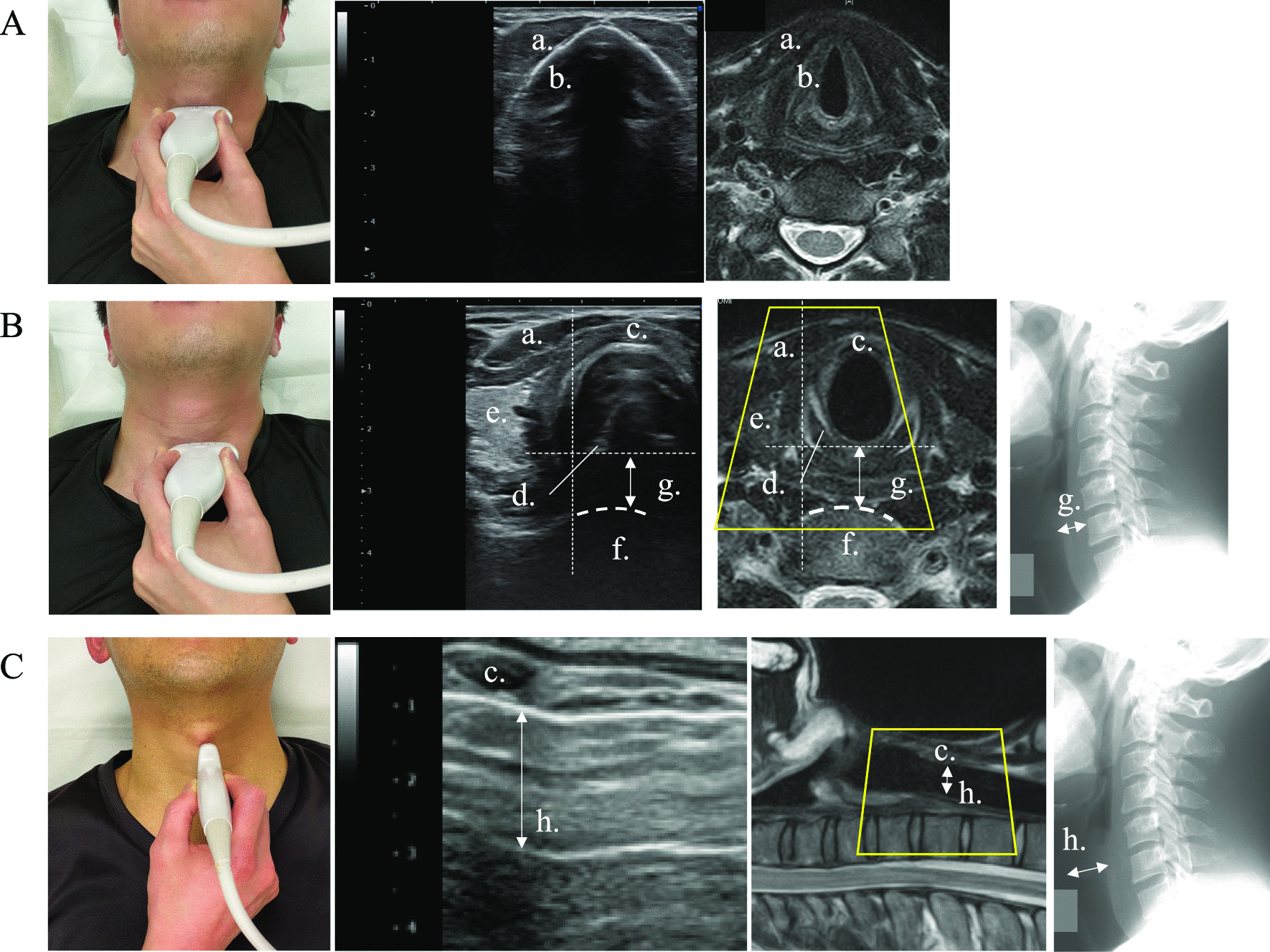
Macro photograph, ultrasound image, and magnetic resonance image **A**, **B**: Thyroid cartilage and cricoid cartilage in the transverse plane. **C**: Cricoid cartilage and tracheal cartilage in the sagittal plane a: sternohyoid muscle, b: thyroid cartilage, c: cricoid cartilage, d; arytenoid cartilage, e: thyroid gland, f: C6 vertebral body, g: the thickness of the prevertebral soft tissue, h: upper airway

The thyroid cartilage, laryngeal prominence, and cricoid cartilage were palpated in the anterior neck region (Fig. 1b, c). The probe was horizontally placed above the thyroid cartilage to visualize the anterior surface in the transverse plane; in the resultant ultrasound image, the thyroid cartilage had a triangular shape (Fig. 2A-b). The probe was then moved to the caudal side, and an arcuate area of the cricoid cartilage was observed on the caudal side of the cricothyroid ligament (Fig. 2B-c). By sliding the probe slightly outward, the anterior surface of the vertebral body was confirmed, and the thickness of the PST was evaluated (Fig. 2B-g). Sliding the probe cranio-caudally allowed detection of the cranio-caudal expansion of the PST.

**Fig. 3:**
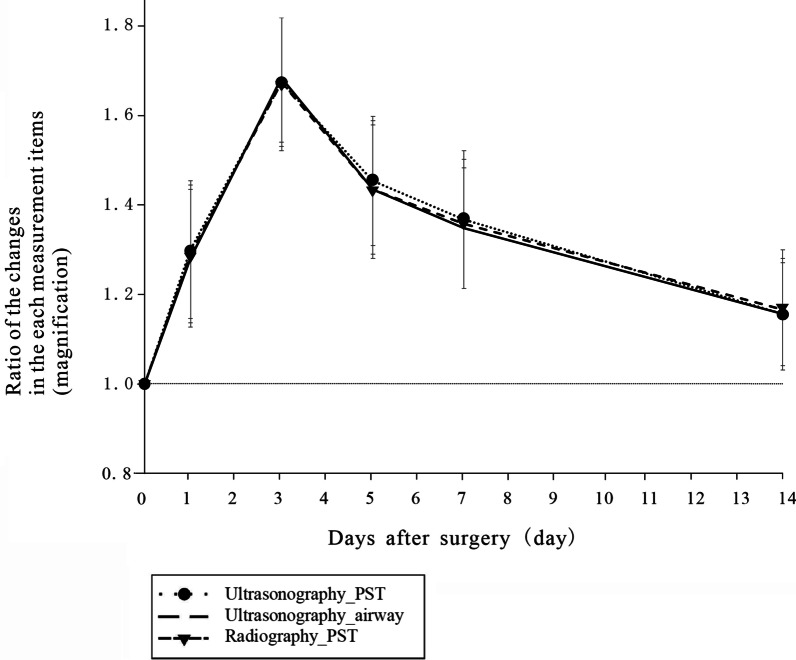
Thickness of the PST and airway in the pilot study. The data are expressed as mean ± standard deviation. PST: prevertebral soft tissue

**Fig. 4:**
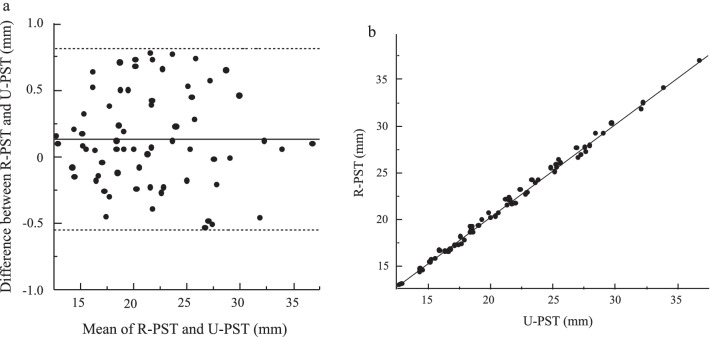
Statistical analysis results of the pilot study. **a**: Bland–Altman plot for mean thickness of prevertebral soft tissue (PST) obtained using radiography versus ultrasonography. **b**: Relationship between PST thickness obtained using radiography (R-PST) and ultrasonography (U-PST) (r = 0.9, *P* < 0.0001)

### Cricoid cartilage and tracheal cartilage in the sagittal plane

The probe was placed sagittally in the anterior neck region to visualize the thyroid cartilage and oval cricoid cartilage with acoustic shadows. Sliding the probe caudally allowed observation of the tracheal cartilage and tracheal surface (Fig. 2C). The boundary between the air in the trachea and anterior wall of the trachea was linearly hyperechoic.

### Outcome measurements

The condition of the PST and upper airway was evaluated consecutively from day 0 (one day before operation) to postoperative day 14. The thickness of the PST at the C6 vertebral body was first measured on a lateral radiograph of the cervical spine and then in a cross section of the cricoid cartilage on the ultrasound image (Fig. 2B). The thickness of the upper airway was assessed in the sagittal plane of the cricoid cartilage and tracheal cartilage via ultrasonography (Fig. 2C). To compensate for differences in the physique of the patients, changes in the thickness of the PST and upper airway over time were normalized to the preoperative thickness, referenced as value “1.”

The PST was measured by an orthopedic surgeon (S.M.) with experience of interpreting spinal radiograph and ultrasound images. To evaluate intraobserver variability, 30 randomly selected ultrasound images were rescored by the same observer (S.M.) more than 1 month after the first reading. Furthermore, to evaluate interobserver variabilities, another 30 randomly selected ultrasound images were examined and scored by a different orthopedic surgeon (H.I.) with experience of interpreting spinal radiograph and ultrasound images. The intraobserver and interobserver variabilities for measurement of PST in ultrasound images evaluated by intraclass correlation coefficient analysis were 0.96 and 0.91, respectively, and were deemed sufficient for assessment.

### Statistical analysis

The Bland–Altman method was used to evaluate the degree of agreement between the PST values obtained using radiography versus ultrasonography [14]. The 95% confidence intervals (CIs) between radiography and ultrasonography were calculated; the clinically acceptable limit was set at ± 1.96 times the standard deviation, which is the upper limit for interscan measurement variability and the threshold for a clinically significant change [8, 15–17]. The Pearson correlation coefficient was used to measure the relationship between the PST values according to the method (radiography versus ultrasonography).

All statistical analyses were performed using JMP version 14 software (SAS Inc., Cary, NC, USA). *P* values < 0.05 were defined as significant.

## Results

### Demographic data of the patients and outcomes

The mean age of the 11 patients in our study was 66.5 years (range, 36–82 years) (Table 1). Six patients were men and five were women. Single-level ACDF was performed in eight cases and double-level ACDF in three. The mean surgical time was 75.9 ± 18.0 min, the mean blood loss was 37.9 ± 10.7 ml, the mean duration of drainage after surgery was 41.6 ± 3.4 h, and the mean amount of drainage was 43.5 ± 16.3 ml.

**Table 1:** Demographic data of the 11 study participants

Age (years)	66.5 ± 16.5 (range 36–82)
Sex (male:female)	6:5
Number of fusion segments	1.27 ± 0.47
Surgical time (minutes)	75.9 ± 18.0
Blood loss (ml)	37.9 ± 10.7
Duration of drainage (hr.)	41.6 ± 3.4
Amount of drainage (ml)	43.5 ± 16.3

The data are expressed as mean ± standard deviation

The mean thickness of the PST (measured via ultrasonography and radiography) and upper airway (measured via ultrasonography) peaked on postoperative day 3 and declined thereafter (Fig. 3). There were no airway complications in these patients. The Bland–Altman bias was 0.13 ± 0.36 mm (95% CI [0.04, 0.22]), which is within the prespecified clinically nonsignificant range. Inspection of the Bland–Altman plot (Fig. 4A) revealed no proportional or systematic errors. The Pearson correlation coefficient (0.99, *P* < 0.0001) indicated a strong correlation between the PST values obtained via radiography and ultrasonography (Fig. 4B)

### Representative case

**Fig. 5:**
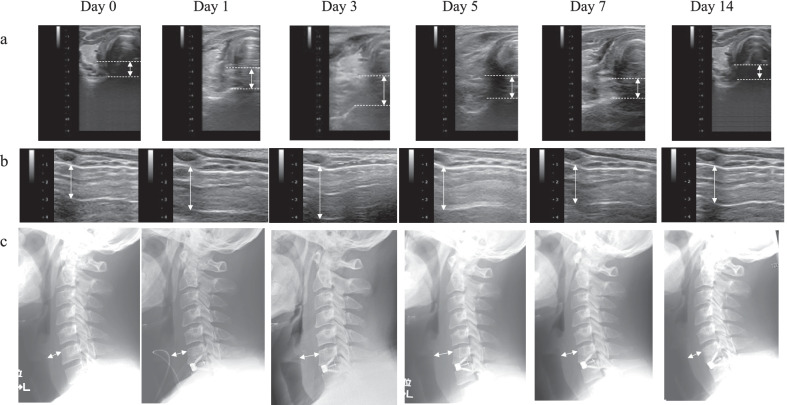
Postoperative course of the prevertebral soft tissue (PST) and upper airway in the representative case. **a**: Postoperative changes in the thickness of the PST measured via ultrasonography. **b**: Postoperative changes in the upper airway measured via ultrasonography. **c**: Postoperative changes in the thickness of the PST measured via lateral radiography of the cervical spine

**Fig. 6:**
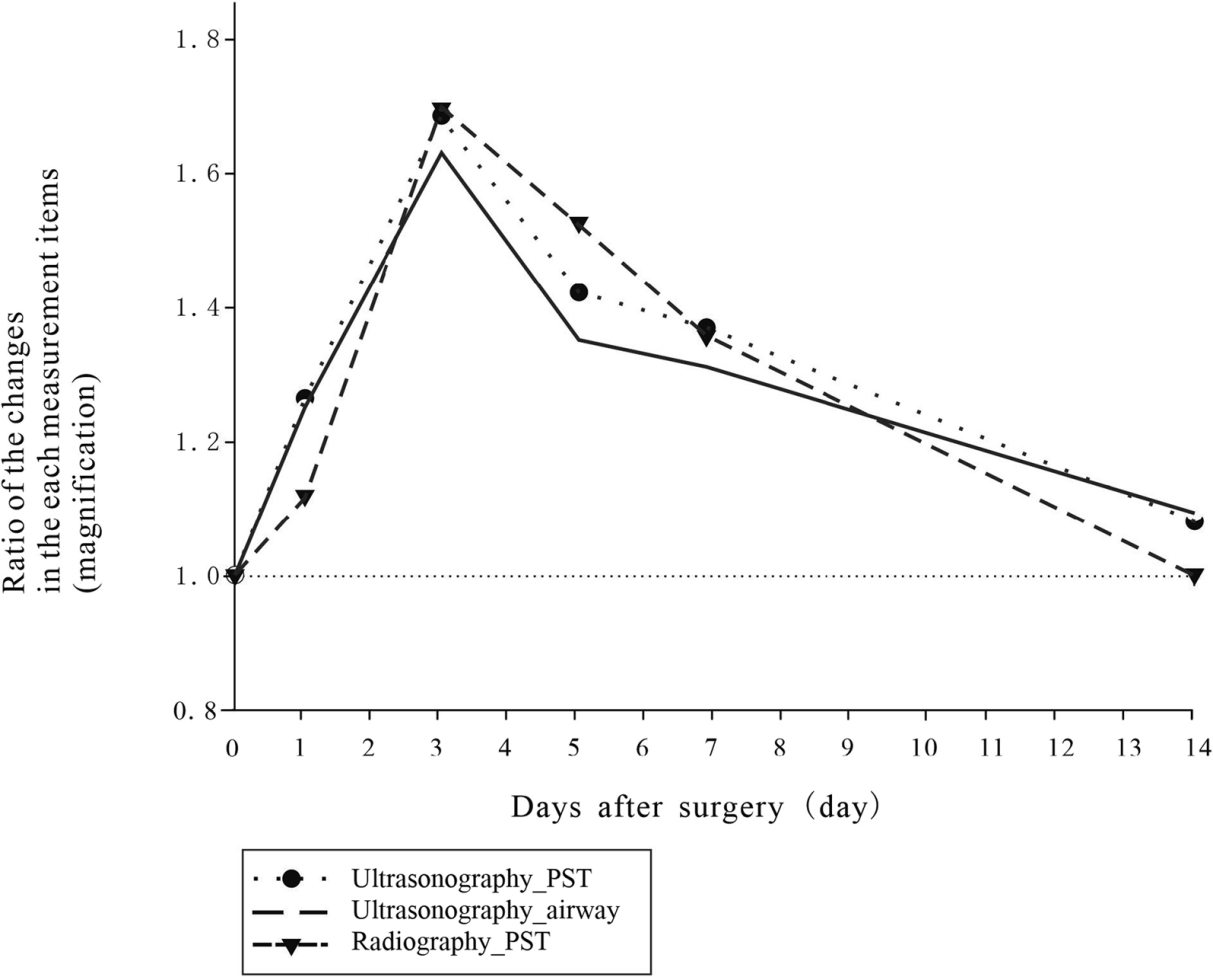
Summary of the information presented in Fig. 4. It can be seen that ultrasonography (upper airway) or both lateral radiography of the cervical spine and ultrasonography (prevertebral soft tissue ) followed a similar course. PST: prevertebral soft tissue

A 36-year-old man presented with right arm pain. Based on the radiological tests and the presence of selective nerve root blockage, the condition was diagnosed as right C6 radiculopathy caused by cervical disc herniation. ACDF for C6/7 cervical disc herniation was performed. Thickness was evaluated consecutively from day 0 (one day before operation) to postoperative day 14 via ultrasonography (upper airway) or both lateral radiography of the cervical spine and ultrasonography (PST). The thickness of the PST peaked on day 3 and gradually decreased after day 5 (Fig. 5A), as did the edema of the upper airway (Fig. 5b). Using ultrasound, it was possible to capture an image of the airway edema, which is difficult to evaluate radiographically (Fig. 5B). Lateral radiographs of the cervical spine measured the PST thickness at the C6 vertebral body (Fig. 5C), showing a very similar course to the ultrasound-measured PST. Figure 6 summarizes the information presented in Fig. 5, showing that ultrasonography (upper airway) or both lateral radiography of the cervical spine and ultrasonography (PST) detected a similar course.

## Discussion

Airway complications are the most serious complications after ACDF and can have devastating consequences if their detection and intervention are delayed [1, 2]. Hence, surgeons must carefully assess the condition of the PST and upper airway by taking lateral radiographs of the cervical spine for several days after surgery. Lateral radiography of the cervical spine has been the mainstay of post-ACDF evaluation. In many hospitals, radiography is routinely used to assess PSTS after ACDF, most commonly by measuring the distance between the anterior margin of the vertebral body and the posterior margin of the airway shadow at the same level [15, 18–21]. PSTS has been reported to peak on postoperative day 3 and gradually decrease after day 5 [15, 21].

Plain radiography is useful for predicting the risk of dyspnea by allowing comparison of the PST thickness before and after surgery. However, it requires frequent radiation exposure and is inconvenient [4, 7–9]. Therefore, we aimed to overcome these problems using ultrasonography to evaluate the PST and upper airway after ACDF. To the best of our knowledge, this is the first report to present a procedure for doing so. In the representative case and pilot study, ultrasonography captured temporal post-ACDF changes in the thickness of the PST and upper airway as efficiently as radiography without exposing the patient to radiation. Ultrasonography could also detect airway edema, which is difficult to evaluate radiographically. Collectively, these results suggest that ultrasonography is more clinically useful to evaluate the PST than radiography from the viewpoint of invasiveness and convenience.

This study has some limitations. First, we were unable to completely replace radiography (the conventional procedure) with ultrasonography because ultrasonography is relatively new, and the data are insufficient for clinical application. Second, our sample size was small. To gather enough data, different cervical levels, numbers of surgical segments, and surgical procedures need to be investigated in a larger sample size. Third, like radiographic evaluation, ultrasound evaluation has no index for comparing measured values among patients. Because patients differ in their physique and soft tissue thickness, comparison with their preoperative condition is necessary. The cut-off value for change between preoperative condition and postoperative condition requiring therapeutic intervention (e.g., steroid administration or tracheal intubation) remians to be clarified in future studies. Fourth, the experience of the ultrasound operator was not addressed. Future studies should compare the results obtained by different operators with different levels of expertise. Because evaluations are somewhat subjective, calculation of a concordance coefficient for agreement among evaluators, along with a side-by-side radiological evaluation, would be helpful.

## Conclusions

This is the first report to describe a procedure for evaluating the condition of the PST and upper airway via ultrasound. Ultrasonography effectively captured post-ACDF changes in the thickness of the PST and upper airway and detected airway edema. Because it can be performed frequently and easily without radiation exposure, it is extremely useful for careful and continuous assessment of the postoperative condition of the PST and upper airway. We suggest that ultrasonography is more clinically useful to evaluate the PST than radiography from the viewpoint of invasiveness and convenience.

## Data Availability

The datasets generated during and/or analyzed during the current study are available from the corresponding author on reasonable request. This is not to restrict the use of our dataset, but to allow the Research Ethics Committee of Wakayama Medical University to understand the actual use of the dataset.

## Abbreviations

ACDF: Anterior cervical decompression and fusion
PST: Prevertebral soft tissue
PSTS: Prevertebral soft tissue swelling
CI: Confidence interval
